# Contributors to caregiver burden, depression, and anxiety in the partners of professional American-style football players: a cross-sectional study

**DOI:** 10.3389/fpsyg.2025.1581239

**Published:** 2025-05-07

**Authors:** Niki Konstantinides, Paula S. Atkeson, Heather DiGregorio, Inana Dairi, Cheyenne Brown, Kairi Noriega, Jillian Baker, Valencia Taylor, Christy Glass, Lewis E. Kazis, Rachel Terrill, Frank E. Speizer, Ross D. Zafonte, Herman A. Taylor, Aaron L. Baggish, Marc G. Weisskopf, Alicia J. Whittington, Rachel Grashow

**Affiliations:** ^1^Football Players Health Study at Harvard University, Harvard Medical School, Boston, MA, United States; ^2^Department of Environmental Health, Harvard T.H. Chan School of Public Health, Boston, MA, United States; ^3^Department of Epidemiology, University of Michigan, Ann Arbor, MI, United States; ^4^Department of Sociology, Utah State University, Logan, UT, United States; ^5^Department of Health Law Policy and Management, Boston University School of Public Health, Boston, MA, United States; ^6^Spaulding Rehabilitation Hospital, Rehabilitation Outcomes Center (ROC), Boston, MA, United States; ^7^Independent Researcher, Indianapolis, IN, United States; ^8^Channing Division of Network Medicine, Brigham and Women's Hospital and Harvard Medical School, Boston, MA, United States; ^9^Department of Physical Medicine and Rehabilitation, Spaulding Rehabilitation Hospital, Charlestown, MA, United States; ^10^Department of Physical Medicine and Rehabilitation, Harvard Medical School, Charlestown, MA, United States; ^11^Cardiovascular Research Institute, Morehosue School of Medicine, Morehouse School of Medicine, Atlanta, GA, United States; ^12^Cardiovascular Performance Program, Massachusetts General Hospital, Boston, MA, United States; ^13^Department of Cardiology, Lausanne University Hospital (CHUV) and Institute for Sport Science, University of Lausanne (ISSUL), Lausanne, Switzerland

**Keywords:** caregiver burden, football (American), depression, anxiety, partners and families

## Abstract

**Introduction:**

American-style football (ASF) has been linked to chronic adverse health outcomes. The extent to which ASF players' careers impact their spouses' caregiver burden, depression, and anxiety remains unknown. In addition to conventional family stressors, ASF families may have specific concerns such as chronic traumatic encephalopathy (CTE; a condition that can only be established at autopsy), which may additionally contribute to caregiver burden and mood symptoms.

**Methods:**

Family Experiences Managing Football Lives (FEM-FL) is a cross-sectional study developed under the Football Players Health Study at Harvard University. Eligible participants were partners of current and former professional ASF players who completed electronic surveys from 2021 to 2024. Data on age, race, family composition, income, employment status, caregiver help, personal health, marital satisfaction, player position, and number of relocations were collected. Participants were asked whether they believed their partner had “CTE.” Multivariable models measured associations between established and ASF-specific risk factors and caregiver burden (Zarit 4-item Burden Interview), and depression and anxiety symptoms (Patient Health Questionnaire-4).

**Results:**

Among 153 partners of active and former professional ASF players, mean [SD] age was 48.1 [13.5], and 28.8% self-identified as Black. In models that adjusted for established risk factors and ASF-specific variables, poor health among partners was associated with a 1.6 point increase in depression score (95% CI = 0.90, 2.30; *p* < *0.001*) and 1.87 point increase in anxiety (95% CI = 1.05, 2.69; *p* < *0.001*). Models that controlled for established risk factors identified significant associations between increased marital satisfaction and a 5.87 reduction in caregiver burden score (95% CI= −7.32, −4.43; *p* < *0.001*), 1.26 score reduction in depression score (95% CI = −1.75, −0.77; *p* < *0.001*) and 1.32 reduction in anxiety score (95% CI = −1.89, −0.75; *p* < *0.001*). CTE concerns were associated with a 2.90 increase in caregiver burden score (95% CI = 1.78, 3.99; *p* < *0.001*) and a 0.44 increase in reported anxiety (95% CI = −0.01, 0.88; *p* = *0.05*), but had no association with depression in adjusted models.

**Discussion:**

Among partners of active and former professional ASF players, marital satisfaction, poor health, and concerns about CTE may play a role in caregiver burden and behavioral health. CTE concerns represents a potential novel risk factor for increased caregiver burden among partners of ASF players.

## Introduction

Conditions and injuries associated with long-term disability and pain affect not only the individual but may also impact family members (Haines et al., 2024; Longo et al., 2020). For example, caregiver burden, defined as “the extent to which caregivers perceived their emotional, physical health, social life, and financial status as a result of caring for their relative” (Zarit et al., 1980) and adverse behavioral and physical health outcomes (Swartz and Collins, 2019) are more prevalent in the partners of individuals with dementia (Besser and Galvin, 2019; Chiari et al., 2021), Alzheimer's Disease (Croog et al., 2006), brain injuries (Brickell et al., 2018; Laratta et al., 2020), and orthopedic injuries (Ziran et al., 2009). Former American-style football (ASF) players represent a population at high-risk of repetitive head (Casson et al., 2010; Pellman et al., 2004) and orthopedic injuries (Chambers et al., 2017; Dodson et al., 2016) that have been linked to neuropsychiatric disease (Roberts et al., 2019), cardiovascular disease (Grashow et al., 2023b), and arthritis (Grashow et al., 2023a). However, the burden that caring for these players places on their long-term partners or spouses has not been investigated. Key risk factors for caregiver burden, depression, and anxiety in the general population include age (Tsai et al., 2021), marital satisfaction (Tough et al., 2017), income (Chou, 2000), family size (Liu et al., 2020), and caregiver health (Bekdemir and Ilhan, 2019; Chou, 2000). Caregiver burden in general populations is associated with (Unsar et al., 2021), although not identical to (Liu et al., 2017; Zhu and Jiang, 2018), mood symptoms like depression and anxiety.

Professional ASF players' experiences may also present unique stressors on their partners. For example, field position has been associated with differences in injury (Casson et al., 2010; Pellman et al., 2004) early aging (Grashow et al., 2023a), and has been shown to track by race (Marquez-Velarde et al., 2023), all of which may be related to caregiver burden, depression and anxiety. During active play, players and their families may experience numerous relocations, uprooting social ties, employment opportunities, and more. Finally, exposure to repetitive head injury has been linked to the neuropathological entity known as chronic traumatic encephalopathy (CTE) in deceased former ASF players (Daneshvar et al., 2023; McKee et al., 2023; Mez et al., 2017), yet has not been definitively linked to clinical premorbid symptoms. Despite being an autopsy-based diagnosis, mainstream media presentations and high-profile cases related to those diagnosed postmortem with CTE may have raised concerns among living players about CTE (Walton et al., 2022) and its research-based clinical correlate, traumatic encephalopathy syndrome (TES) (Katz et al., 2021). Given that two recent studies showed that ~35% of former players have concerns about CTE (Grashow et al., 2024; Walton et al., 2022), there may be a sizable number of families with concerns about long-term brain health.

Established and ASF-specific determinants of caregiver burden, depression, and anxiety among partners of ASF players have been incompletely characterized. The objectives of this study were to: (1) describe the Family Experiences Managing Football Lives (FEM-FL) cohort, a substudy of the Football Players Health Study at Harvard University (Zafonte et al., 2019) recruiting partners and spouses of current and former ASF players; (2) identify associations between established risk factors and ASF partner caregiver burden, depression, and anxiety; and (3) measure the extent to which ASF-specific stressors impact the outcomes of interest after adjusting for established risk factors. We hypothesized that established risk factors for caregiver burden would be relevant in a cohort of ASF partners and spouses, and that football-specific stressors would independently be associated with caregiver burden.

## Materials and methods

### Study participants

The Family Experiences Managing Football Lives (FEM-FL) study stands as an independent separate cohort that investigates the impact of ASF careers on spouses' physical, mental, and emotional health. Anyone currently partnered with an active or former ASF player was considered eligible. Both FPHS and FEM-FL utilize the community-based participatory research model (CBPR; Viswanathan et al., 2004) that prioritizes collaboration with the ASF family community, ASF partner organizations, clinicians, and researchers (Viswanathan et al., 2004) in study design, recruitment, and result presentation.

### Recruitment

In accordance with CBPR principles, FEM-FL created a FEM-FL Advisory Board comprised of physicians, organizational leaders, and community members who were spouses and family members of ASF players. Recruitment was conducted in-person and remotely with promotional FEM-FL materials. Currently, there is no centralized list of partners and wives of active and former players. We therefore recruited through the Football Players Health Study at Harvard University (FPHS) (Zafonte et al., 2019) which was designed to investigate the health and wellbeing of former professional ASF players. Therefore, to maximize outreach to community members, FEM-FL invitations were printed in ASF family community publications, posted on social media, sent electronically to FPHS participants, and included in ASF community organization outreach. To be eligible for this study, participants had to be currently partnered with a former or active ASF player. There were no other exclusion criteria for recruitment. One hundred seventy-two participants enrolled in FEM-FL between February 2021 and March 2024. The study was approved by the Institutional Review Board of the Harvard T.H. Chan School of Public Health, and participants provided informed consent prior to enrollment. All research practices were performed in accordance with the Declaration of Helsinki.

### Demographic measures

All FEM-FL participants identified as female. Demographic data were collected, including age, and self-identified race (Black/African American, White/Caucasian, American Indian/Alaskan Native, Native Hawaiian/Pacific Islander, Asian, Other, and missing). Body mass index (BMI) at the time of survey completion was calculated from self-reported weight (pounds) and height (inches). Alcohol consumption per week was calculated from: “In a typical week, how many days do you drink a beverage containing alcohol?” and “On a typical day that you drink, how many beverages containing alcohol do you usually have?” We queried the women on current smoking status. Questions on caregiver burden, participant depression, and participant anxiety were included. To determine marital satisfaction, participants were asked, “How satisfied are you with your marriage or romantic partnership?” with the option to select, “Very satisfied,” “Moderately satisfied,” “Slightly satisfied,” “Neutral,” “Slightly dissatisfied,” “Moderately dissatisfied,” or “Very dissatisfied.” Answers were categorized as “satisfied” and “dissatisfied”, “neutral” was included in “satisfied.” Approximate household net worth was classified as less than $5,000, $5,000–$49,000, $50,000–$169,000, $170,000–$499,000, $500,000–$999,000 and $1,000,000+, and then categorized into “$169,000 or less” and “$170,000 or more.” The question about health was phrased as “In general, would you say your health is…” with the option to select: “Excellent,” “Very good,” “Good,” “Fair,” or “Poor.” Answers were dichotomized into “Has poor health” and “Does not have poor health,” “Fair” was included in “Does not have poor health.” Participants were asked, “Which term best describes your primary work role?” with the option to select: “Employed for wages,” “Self-employed,” “Out of work and looking for work,” “Homemaker,” “Student,” “Military,” “Retired,” or “Unable to work.” Answers were dichotomized as “salaried worker” and “non-salaried” (i.e., out of work and looking for work, homemaker, retired, and unable to work). Lastly, for caregiver help, participants were asked “Do you currently employ or utilize any of the following for help with your partner, children, or older adults?” with the option to select all that applied: nanny, home health aide, help from another relative, other, and none. Answers were dichotomized as either “yes have help” or “no help.”

### ASF-specific variables

Partners' ASF position was determined by asking, “To the best of your knowledge, what position did your partner primarily play while in the NFL?” Answers were dichotomized as either lineman or non-lineman. Number of relocations were asked with the phrasing, “How many times did you relocate based on your partner's NFL career?” Answers were dichotomized as being either below or equal to the mean, or above the mean. To assess participant CTE concerns, the survey asked “Do you believe your partner has Chronic Traumatic Encephalopathy?” No definition of CTE was provided for either question due to lack of clinical consensus on pre-mortem CTE (Lenihan and Jordan, 2015; Randolph, 2018; Taghdiri et al., 2024).

### Outcome variables

The four-item Zarit Burden Interview (ZBI-4) assessed feelings of caregiver burden when supporting a partner's health (Higginson et al., 2010). Responses to the ZBI-4 include, “Never,” “Rarely,” “Sometimes,” “Often,” and “Nearly always.” Lower caregiver burden scores reflect less perceived stress associated with partner care. Scores may range from 0–16, and a score of eight is considered to reflect “severe” caregiver burden (Bédard et al., 2001). Depression and anxiety symptom severities over the past two weeks were assessed using the two-item Patient Health Questionnaire (PHQ-2; Löwe et al., 2005) and two item Generalized Anxiety Disorder (GAD-2; Delgadillo et al., 2012), respectively. Both the PHQ-2 and GAD-2 responses include “Not at all,” “Several days,” “More than half the days,” and “Nearly every day.” In the PHQ-2 and GAD-2, scores may range from 0–6, and a score of three is considered to be clinically depressed and clinically anxious, respectively (Staples et al., 2019).

### Statistical analysis

Only participants who completed questions on the outcomes of interest (caregiver burden, depression, and anxiety) were included in analyses (*N* = 153). Bivariate associations were calculated between demographic, lifestyle, and current health variables and outcomes of concern [caregiver burden (less than eight, greater than or equal to eight), depression symptoms (less than three, greater than or equal to three), and anxiety symptoms (less than three, greater than or equal to three)] using Kruskal-Wallis rank sum tests for continuous variables and chi-square tests for categorical variables. For one participant missing age, age was imputed using multiple imputation by chained equations (MICE; Buuren and Groothuis-Oudshoorn, 2011) based on race, marital satisfaction, income, health, and having young children. Race was categorized as Black, White, and Other. Welch Two-Sample *t*-tests determined differences between levels of each predictor for caregiver burden, depression, and anxiety outcomes. We used linear regression to estimate associations and 95% confidence intervals between each risk factor and the three outcomes separately. Initial models adjusted only for demographics (age and race). Any established risk factor associated with any outcome of interest was included in final models that adjusted for ASF-specific exposures. To correct for multiple comparisons, we implemented a Benjamini-Hochberg correction in analyses that included multiple hypothesis testing (Benjamini and Hochberg, 1995). A *post hoc* power analysis was conducted for a sample size of 153 with an alpha level of 0.05. Based on these parameters, the minimum effect size (caregiver burden score) we had 80% power to detect was 0.95. Statistical significance was considered at *p* < 0.05 except where noted, and analyses were conducted using R Language for Statistical Computing (R Core Team, 2022).

## Results

Among 172 FEM-FL participants (Supplementary Table 1), 153 completed all data on caregiver burden, depression, and anxiety (Table 1). The average ± SD age of the FEM-FL analytic cohort was 48.1 ± 13.5 years, and 93 self-identified as white (60.8%). Two participants reported being partnered to a current ASF player. Eighty-one (52.9%) participants reported body mass index (BMI) < 25.0, 82 (53.6%) reported not drinking alcohol, three (1.9%) reported current tobacco smoking, and 119 (77.77%) earned a bachelor's degree or higher. One hundred twenty-two (79.7%) reported marital satisfaction, and 14 (9.2%) reported having poor health. Among the 153 participants who provided data on CTE concerns, 60 (41.7%) endorsed believing their player-partner had CTE.

**Table 1:** Characteristics of the FEM-FL cohort with complete questions on caregiver burden, depression, and anxiety.

**Variable**	**Overall (*N* = 153)**
**Age**	
Mean (SD)	48.10 (13.5)
**Race**	
Black	44 (28.8%)
White	93 (60.8%)
Other	13 (8.5%)
Missing, *N*	3 (2.0%)
**Partner current stage**	
Active player	2 (1.31%)
Post-career (0–5 years)	19 (12.41%)
Post-career (6 or more years)	126 (82.35%)
Missing, *N*	6 (3.93%)
**Domestic status**	
Married	138 (90.1%)
Living with partner	7 (4.6%)
Other	7 (4.6%)
Missing, *N*	1 (0.7%)
**Current BMI**	
< 25.0	81 (52.9%)
25.0–30.0	47 (30.7%)
>30.0	22 (14.4%)
Missing, *N*	3 (2.0%)
**Drinks per week**	
None	82 (53.6%)
1–7 drinks/week	53 (34.6%)
8–14 drinks/week	10 (6.5%)
15+ drinks/week	7 (4.6%)
Missing, *N*	1 (0.7%)
**Smoking status**	
Never smoked	131 (85.6%)
Quit or stopped smoking	19 (12.4%)
Current smoker	3 (1.9%)
**Highest level of education**	
Graduated high school	7 (4.6%)
Some college	14 (9.2%)
Associate's degree	8 (5.23%)
Bachelor's degree	67 (43.7%)
Master's degree	41 (26.8%)
Medical or doctoral degree	11 (7.2%)
Missing, *N*	5 (3.27%)
Non-lineman	98 (64.1%)
Lineman	55 (35.9%)
**Number of relocations**	
Mean (SD)	1.7 (2.1)
Missing, *N*	10
Has marital satisfaction	122 (79.7%)
**Income level**	
Less than $169,000	53 (34.6%)
More than $170,000	100 (65.4%)
Has poor health	14 (9.2%)
Has employment	108 (70.6%)
Has young children	58 (37.9%)
Has caregiver help	132 (86.3%)
Participant CTE concern	60 (41.7%)
Missing, *N*	9 (5.88%)
**Caregiver burden total**	
Mean (SD)	5.1 (4.2)
**Participant depression symptoms**	
Mean (SD)	0.9 (1.4)
**Participant anxiety symptoms**	
Mean (SD)	1.3 (1.6)

CTE, Chronic traumatic encephalopathy.

**Figure 1:**
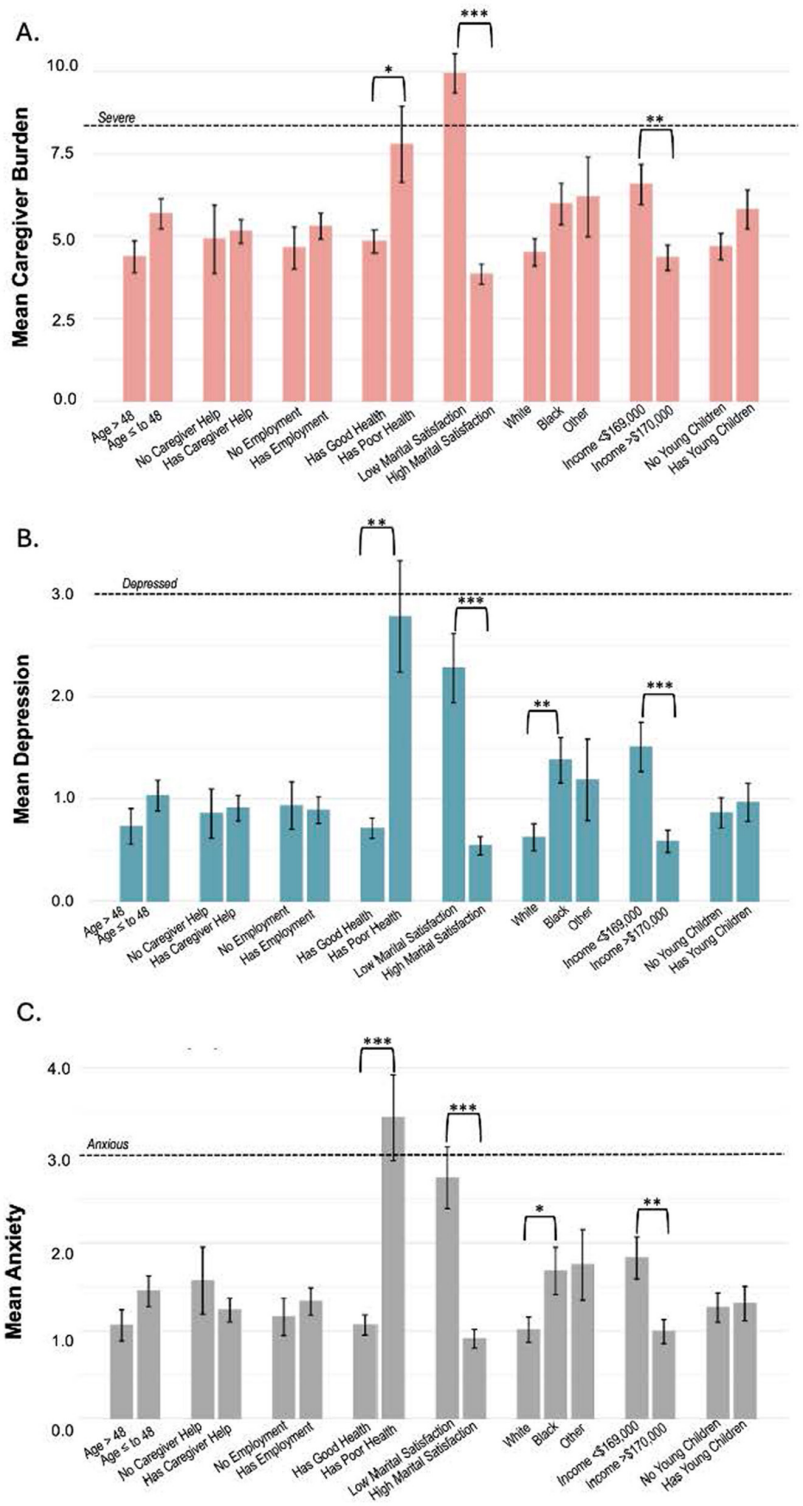
**(A)** Caregiver burden, **(B)** depression, and **(C)** anxiety vs. demographic, health, and ASF-related characteristics. The dotted lines represent **(A)** a score of 8, which is defined as “severe” caregiver burden, **(B)** a PHQ-2 score of three in which an individual is considered “depressed,” and **(C)** a GAD-2 score of three in which an individual is considered to be “anxious.” *p < 0.05, **p < 0.01, ***p < 0.001.

Bivariate associations between demographic and family characteristics are shown in Figure 1. Marital satisfaction and race (white vs. Black) were associated with all three outcomes (Figures 1A–C). Income was significantly associated with caregiver burden (Figure 1A) and anxiety (Figure 1C), and poor health was significantly associated with depression (Figure 1B) and anxiety (Figure 1C).

We first investigated demographic (adjusted for age and race) models treating a single established risk factor as an independent variable and found that they were significantly associated with the three outcomes. In these age- and race-adjusted models for each non-football factor we saw significant associations between race, wealth, health, and marital satisfaction only (Supplementary Table 1). We then ran models that predicted each of the three outcomes of interest using all established risk-factors in the same model (Supplementary Table 2). The significant contributors from these models (age, poor health and marital satisfaction; Supplementary Table 2) were then incorporated into models with ASF-specific risk factors (number of relocations, and CTE concerns).

**Figure 2:**
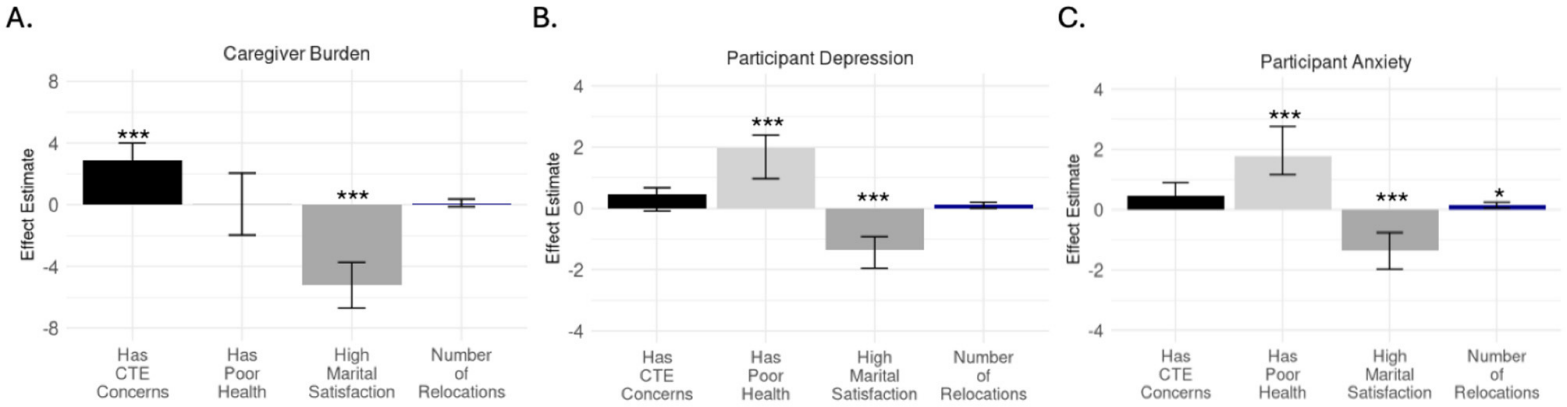
Effect estimates for **(A)** caregiver burden, **(B)** participant depression and **(C)** participant anxiety from models additionally adjusted with age and ASF-specific variables, such as lineman status, number of relocations, and CTE concerns. These analyses have been adjusted for multiple comparisons using Benjamini-Hochberg method. Parameter estimates that were significantly associated with any outcome are shown, with any outcome are shown, with output on all variables available in Supplementary material. *p < 0.05, ***p < 0.001. CTE, chronic traumatic encephalopathy.

In models adjusted for age, poor health and marital satisfaction, CTE concerns were significantly associated with a 2.90 higher caregiver burden score (95% CI = 1.78, 3.99; *p* < *0.0001*; Figure 2A; Supplementary Table 3), and 0.44 higher anxiety score (95% CI = −0.01, 0.88; *p* = *0.05*; Figure 2C; Supplementary Table 3). CTE concerns were not significantly associated with depression or anxiety (Figure 2B; Supplementary Table 3). Partner lineman status showed no significant association with caregiver burden, depression, or anxiety in adjusted models (Supplementary Table 3); however, number of relocations was significantly associated with slightly increased anxiety (Figure 2C; Supplementary Table 3).

## Discussion

To our knowledge, FEM-FL is the first study of the spouses of active and former professional ASF players to investigate established and ASF-specific contributors to caregiver burden, depression, and anxiety in partners of ASF players. In fully adjusted models, marital satisfaction, poor health, and CTE concerns were associated with at least one of the outcomes of interest. Although CTE concerns were significantly associated with increased caregiver burden and anxiety, there was no association with depression. Although, among prior ASF players, CTE concern is likely inevitable due to the highly publicized link between ASF participation and subsequent brain disease (Bracken et al., 2023; Convery, 2023; Schwarz, 2007; Shpigel, 2022; Walton et al., 2022). This is the first study to investigate how the partners of these players may be affected by such concerns.

Previous studies have shown that caregiver burden is associated with negative mental and physical health outcomes in those with conditions such as dementia (Fauth et al., 2012; Schneider et al., 2002), stroke (Visser-Meily et al., 2004), cancer (Goldstein et al., 2004), and others (Janson et al., 2022). In a study of traumatic brain injury (TBI) patients, caregivers similarly experienced caregiver overload, depression, and anxiety (Mena-Marcos et al., 2024). Findings from this study suggest that caregiver burden is higher in participants who reported concerns about CTE their partners compared to those who do not. Interestingly, in this study, average total burden scores for participants with CTE concerns (7.5) were greater than averages measured using the ZBI-4 in caregivers of older family members (6.3; Alves et al., 2022), those with Parkinson's Disease (5.5; Hagell et al., 2017), dementia (6.1; Higginson et al., 2010), cancer (6.4; Higginson et al., 2010), and slightly less than acquired brain injury (7.9; Higginson et al., 2010). However, in a study of partners in a military household, the median ZBI-4 score was 10 (Shepherd-Banigan et al., 2020), approximately equivalent to participants with CTE concerns (median ZBI-4 = 9). Average depression scores (0.9) measured with the PHQ-2 were similar to scores reported by caregivers of dementia patients (1.1; Smolcic et al., 2016), and non-caregivers (0.97; Wicke et al., 2022), (1.0; Kroenke et al., 2009), but lower than those reported in caregivers of cancer patients (1.82; Sklenarova et al., 2015). Average anxiety scores measured by the GAD-2 (1.3) were similar to anxiety scores reported by cohorts of general participants (0.83; Wicke et al., 2022), (1.4; Kroenke et al., 2009), and less than reported scores from caregivers of cancer patients (2.15; Sklenarova et al., 2015). Amongst our cohort, depression and anxiety scores on average increased due to participant poor health and low marital satisfaction, reaching similar scores reported by caregivers of cancer patients (Sklenarova et al., 2015). These results may suggest that caregiver burden magnitude remains consistently high in partners of active and former ASF players and may approximate that of caregivers within military families, a population also at risk for repetitive head and orthopedic trauma. In contrast, depression and anxiety scores were on average close to scores reported by non-caregivers, but may be more significantly associated with other lifestyle factors.

After adjusting for ASF-specific characteristics in a model with significant established risk factors, number of relocations was significantly associated with depression and anxiety. CTE concerns among ASF partners were significantly associated with caregiver burden and anxiety, but not depression. Although tau-deposition consistent with CTE is a common autopsy finding among prior professional ASF players, current pre- and post-mortem tau deposition studies have not been definitively linked to proposed clinical attributes, such as depression, anxiety, suicidality, and cognitive dysfunction (Iverson et al., 2023; Mez et al., 2021; Stern et al., 2019). However, ASF players and their partners may experience concerns about CTE due to cognitive symptoms not attributed to other health conditions, and to mainstream media reports that implicate CTE as the underlying cause of neurocognitive dysfunction and psychiatric symptoms among former professional ASF players (Bracken et al., 2023; Convery, 2023; Schwarz, 2007; Shpigel, 2022). In a study of former ASF players, 39.5% of participants reported being “currently extremely concerned” about CTE (Walton et al., 2022), similar to another study which found 34.4% reported believing they have CTE (Grashow et al., 2024). This illustrates that although there is no direct pathophysiological mechanism linking previous football play and/or injuries sustained during football play with CTE, many athletes may perceive this risk. These numbers are approximately similar in that we identified that 41.2% of participants held concerns that their player partner had CTE.

This study has several limitations. First, these data were all self-reported by participants. Second, we cannot determine whether participant CTE concerns are due to the effects of underlying neuropathology, a misattribution of symptoms caused by alternative disease processes, another factor, or some combination. This represents an area of important future work which will only be possible when accurate tools to diagnose CTE in living players have been developed. Third, we do not have data on other conditions or symptoms the partner of the participant has been diagnosed with or is currently experiencing. Data from other studies have shown that those who reported being diagnosed with pre-mortem CTE (Grashow et al., 2020) were more likely to also report conditions associated with cognitive symptoms such as sleep apnea, low testosterone, hypertension, pain, and more. Fourth, we also have no way of determining whether the results described here can be generalized to the entire NFL partner population because there is currently no way to identify and reach all former and active ASF player spouses. We therefore acknowledge the possibility of a biased sample and that our findings may not apply to all partners of former and current ASF players. Finally, since this study only examined partners of former ASF players, results may not be generalizable to the partners of athletes of other professional sports.

In conclusion, one-third of partners of active and former professional ASF players report concerns about CTE. Data from this study established an association between participant CTE concerns and caregiver burden and anxiety. More research is needed to understand the risks associated with CTE concerns not only in former and current ASF players, but also in their partners and families.

These results should motivate future work that investigates the factors associated with concerns about CTE in former and active player's partners, and what interventions may be implemented to reduce caregiver burden and support the physical and mental health of partners and families of professional ASF players.

## Data Availability

The datasets presented in this article are not readily available because participant survey responses used in this study could be used to recognize the identities of participants, and are therefore under the protection of a Certificate of Confidentiality granted from the NIH. Questions regarding the datasets should be directed to rgrashow@hsph.harvard.edu.
