# Supplementary material for: Contributors to caregiver burden, depression, and anxiety in the partners of professional American-style football players: a cross-sectional study
Source: Front Psychol. 2025 May 7;16:1581239. doi: 10.3389/fpsyg.2025.1581239 (PMC12092435; doi:10.3389/fpsyg.2025.1581239)
Supplement: Supplementary file 1 [file Table_1.docx]

Supplementary Material

**eTable 1:** Characteristics of the FEM-FL cohort.

| **Variable** | **Overall (N=172)** |
| --- | --- |
| **Age** |  |
| Mean (SD) | 48.0 (13.0) |
| Missing, N | 6 |
| **Race** |  |
| Black | 50 (29.1%) |
| White | 103 (59.9%) |
| Other | 15 (8.7%) |
| Missing, N | 4 (2.3%) |
| **Partner Current Stage** |  |
| Active Player | 2 (1.2%) |
| Post-career (0-5 years) | 20 (11.6%) |
| Post-career (6 or more years) | 134 (77.9%) |
| Missing, N | 16 (9.3%) |
| **Domestic Status** |  |
| Married | 152 (88.38%) |
| Living with partner | 10 (5.81%) |
| Other | 7 (4.07%) |
| Missing, N | 3 (1.74%) |
| **Current BMI** |  |
| <25.0 | 83 (48.3%) |
| 25.0-30.0 | 50 (29.1%) |
| >30.0 | 27 (15.7%) |
| Missing, N | 12 (7.0%) |
| **Drinks Per Week** |  |
| None | 91 (52.9%) |
| 1-7 drinks/week | 60 (34.9%) |
| 15+ drinks/week | 7 (4.1%) |
| 8-14 drinks/week | 11 (6.4%) |
| Missing, N | 3 (1.7%) |
| **Smoking Status** |  |
| Never smoked | 143 (83.15%) |
| Quit or stopped smoking | 24 (13.95%) |
| Current smoker | 3 (1.74%) |
| Missing, N | 2 (1.16%) |
| **Highest Level of Education** |  |
| Graduated high school | 8 (4.65%) |
| Some college | 15 (8.72%) |
| Associate’s degree | 8 (4.65%) |
| Bachelor’s degree | 71 (41.28%) |
| Master’s degree | 44 (25.58%) |
| Medical or doctoral degree | 11 (6.40%) |
| Missing, N | 15 (8.72%) |
| **Player Position** |  |
| Non-lineman | 115 (66.9%) |
| Lineman | 57 (33.1%) |
| **Number of Relocations** |  |
| Mean (SD) | 1.7 (2.1) |
| Missing, N | 20 |
| **Has Marital Satisfaction** | 130 (75.60%) |
| Missing, N | 15 (8.72%) |
| **Income Level** |  |
| Less than $169,000 | 56 (32.6%) |
| More than $170,000 | 116 (67.4%) |
| **Has Poor Health** | 22 (12.8%) |
| **Has Employment** | 121 (70.3%) |
| **Has Young Children** | 61 (35.5%) |
| **Has Caregiver Help** | 138 (80.2%) |
| **Participant CTE Concern** | 63 (36.62%) |
| Missing, N | 19 |
| **Caregiver Burden Total** |  |
| Mean (SD) | 5.1 (4.2) |
| Missing, N | 15 |
| **Participant Depression Symptoms** |  |
| Mean (SD) | 0.9 (1.4) |
| Missing, N | 11 |
| **Participant Anxiety Symptoms** |  |
| Mean (SD) | 1.3 (1.5) |
| Missing, N | 10 |

CTE=Chronic traumatic encephalopathy

**eTable 2:** Effect estimates*, p-values, and 95% confidence intervals for variables associated with caregiver burden, depression, and anxiety.

|  | **Caregiver Burden** | | | **Depression** | | | **Anxiety** | | |
| --- | --- | --- | --- | --- | --- | --- | --- | --- | --- |
| **Variable** | **Estimate** | **p-value** | **95% CI** | **Estimate** | **p-value** | **95% CI** | **Estimate** | **p-value** | **95% CI** |
| Age | -0.04 | 0.07 | -0.09, 0.004 | -0.01 | 0.10 | -0.03, 0.003 | -0.02 | 0.09 | -0.04, 0.003 |
| Race: Black | 1.25 | 0.10 | -0.24, 2.74 | 0.68 | 0.008 | 0.19, 1.18 | 0.59 | 0.04 | 0.03, 1.15 |
| Race: Other | 1.15 | 0.32 | -1.11, 3.42 | 0.40 | 0.30 | -0.36, 1.16 | 0.55 | 0.20 | -0.29, 1.40 |
| Wealth | -1.81 | 0.01 | -3.22, -0.41 | -0.76 | 0.002 | -1.22, -0.29 | -0.66 | 0.01 | -1.12, -0.14 |
| Has young children | 0.72 | 0.38 | -0.88, 2.33 | -0.08 | 0.76 | -0.62, 0.45 | -0.24 | 0.44 | -0.83, 0.36 |
| Has poor health | 2.39 | 0.04 | 0.08, 4.67 | 1.88 | <0.001 | 1.16, 2.60 | 2.18 | <0.001 | 1.38, 2.97 |
| Has employment | 0.07 | 0.93 | -1.44, 1.57 | -0.34 | 0.21 | -0.83, 0.18 | -0.07 | 0.80 | -0.64, 0.50 |
| Has caregiver help | 0.91 | 0.36 | -1.05, 2.88 | 0.30 | 0.37 | -0.36, 0.95 | -0.08 | 0.82 | -0.82, 0.65 |
| Has marital satisfaction | -6.05 | <0.001 | -7.40, -4.70 | -1.63 | <0.001 | -2.12, -1.14 | -1.74 | <0.001 | -2.30, -1.18 |

CTE = Chronic traumatic encephalopathy

* Models for age and race are adjusted for the other; all other models are adjusted for age and race.

**eTable 3:** Effect estimates, p-values, and 95% CI for established risk factors (Model 1; age, race, established risk factors) and ASF factors (Model 2; significant terms from Model 1 plus lineman status, number of relocations, and CTE concerns). Q-values are adjusted p-values when accounting for multiple comparisons using the Benjamini-Hochberg method.

|  | **Model 1: Established factors model** | | | |  | **Model 2: ASF exposures model** | | |
| --- | --- | --- | --- | --- | --- | --- | --- | --- |
| **Caregiver Burden** | | | | | | | | |
| **Variable** | **Estimate** | **95% CI** | **p-value** | **q-value** | **Estimate** | **95% CI** | **p-value** | **q-value** |
| Age | -0.03 | -0.08, 0.02 | 0.21 | 0.36 | -0.04 | -0.08, -0.01 | 0.02 | 0.06 |
| Race: Black | -0.11 | -1.43, 1.20 | 0.87 | 0.95 | - | - | - | - |
| Race: Other | 0.47 | -1.42, 2.38 | 0.62 | 0.78 | - | - | - | - |
| Wealth | -1.02 | -2.20, 0.16 | 0.09 | 0.19 | - | - | - | - |
| Has young children | 0.60 | -0.73, 1.93 | 0.37 | 0.52 | - | - | - | - |
| Has poor health | -0.22 | -2.24, 1.79 | 0.83 | 0.95 | -0.34 | -2.38,1.70 | 0.74 | 0.85 |
| Has employment | -0.03 | -1.27, 1.22 | 0.97 | 0.97 | - | - | - | - |
| Has caregiver help | 0.55 | -1.06, 2.17 | 0.50 | 0.64 | - | - | - | - |
| High marital satisfaction | -5.87 | -7.32, -4.43 | <0.001 | 0.004 | -5.15 | -6.58, -3.72, | <0.001 | 0.004 |
| Partner Lineman Status | - | - | - | - | -0.16 | -1.24, 0.92 | 0.77 | 0.94 |
| Number of Relocations | - | - | - | - | 0.11 | -0.13,0.36 | 0.37 | 0.52 |
| Has CTE Concerns | - | - | - | - | 2.90 | 1.78, 3.99 | <0.001 | 0.004 |
| **Depression** | | | | | | | | |
| Age | -0.01 | -0.03, 0.004 | 0.13 | 0.23 | -0.01 | -0.03, -0.002 | 0.10 | 0.20 |
| Race: Black | 0.22 | -0.23, 0.67 | 0.33 | 0.51 | - | - | - | - |
| Race: Other | 0.04 | -0.61, 0.69 | 0.90 | 0.97 | - | - | - | - |
| Wealth | -0.57 | -0.97, -0.16 | 0.006 | 0.02 | -0.56 | -0.96, -0.16 | 0.007 | 0.02 |
| Has young children | -0.06 | -0.51, 0.39 | 0.79 | 0.94 | - | - | - | - |
| Has poor health | 1.27 | 0.58, 1.96 | <0.001 | 0.004 | 1.60 | 0.90, 2.30 | <0.001 | 0.004 |
| Has employment | -0.39 | -0.82, 0.03 | 0.07 | 0.16 | - | - | - | - |
| Has caregiver help | 0.03 | -0.52, 0.58 | 0.92 | 0.96 | - | - | - | - |
| High marital satisfaction | -1.26 | -1.75, -0.77 | <0.001 | 0.004 | -1.43 | -1.95, -0.92 | <0.001 | 0.004 |
| Partner Lineman Status | - | - | - | - | -0.34 | -0.72, 0.03 | 0.11 | 0.22 |
| Number of Relocations | - | - | - | - | 0.10 | 0.01,0.19 | 0.03 | 0.08 |
| Has CTE Concerns | - | - | - | - | 0.17 | -0.21, 0.56 | 0.37 | 0.52 |
| **Anxiety** | | | | | | | | |
| Age | -0.02 | -0.03, 0.004 | 0.13 | 0.23 | -0.02 | -0.03, 0.001 | 0.06 | 0.14 |
| Race: Black | 0.02 | -0.50, 0.53 | 0.95 | 0.97 | - | - | - | - |
| Race: Other | 0.07 | -0.67, 0.81 | 0.85 | 0.95 | - | - | - | - |
| Wealth | -0.44 | -0.90, 0.02 | 0.06 | 0.14 | - | - | - | - |
| Has young children | -0.18 | -0.70, 0.34 | 0.49 | 0.64 | - | - | - | - |
| Has poor health | 1.58 | 0.79, 2.37 | <0.001 | 0.004 | 1.87 | 1.05, 2.69 | <0.001 | 0.004 |
| Has employment | -0.17 | -0.66, 0.31 | 0.48 | 0.64 | - | - | - | - |
| Has caregiver help | -0.36 | -1.00, 0.27 | 0.25 | 0.42 | - | - | - | - |
| High marital satisfaction | -1.32 | -1.89, -0.75 | <0.001 | 0.004 | -1.42 | -2.02, -0.82 | <0.001 | 0.004 |
| Partner Lineman Status | - | - | - | - | -0.24 | -0.68, 0.19 | 0.27 | 0.43 |
| Number of Relocations | - | - | - | - | 0.15 | 0.05, 0.25 | 0.003 | 0.01 |
| Has CTE Concerns | - | - | - | - | 0.44 | -0.01, 0.88 | 0.05 | 0.13 |

CTE = Chronic traumatic encephalopathy
